# A polymorphism at the 3'-UTR region of the aromatase gene defines a subgroup of postmenopausal breast cancer patients with poor response to neoadjuvant letrozole

**DOI:** 10.1186/1471-2407-10-36

**Published:** 2010-02-09

**Authors:** Zaida Garcia-Casado, Angel Guerrero-Zotano, Antonio Llombart-Cussac, Ana Calatrava, Antonio Fernandez-Serra, Amparo Ruiz-Simon, Joaquin Gavila, Miguel A Climent, Sergio Almenar, Jose Cervera-Deval, Josefina Campos, Carlos Vazquez Albaladejo, Antonio Llombart-Bosch, Vicente Guillem, Jose A Lopez-Guerrero

**Affiliations:** 1 Laboratory of Molecular Biology, Fundación Instituto Valenciano de Oncología, Valencia, Spain; 2 Department of Medical Oncology, Fundación Instituto Valenciano de Oncología, Valencia, Spain; 3 Department of Medical Oncology, Hospital Arnau de Villanova, Lleida, Spain; 4 Department of Pathology, Fundación Instituto Valenciano de Oncología, Valencia, Spain; 5 Department of Pathology, University of Valencia, Spain; 6 Department of Radiology, Fundación Instituto Valenciano de Oncología, Valencia, Spain; 7 Department of Surgery, Fundación Instituto Valenciano de Oncología, Valencia, Spain

## Abstract

**Background:**

Aromatase (*CYP19A1*) regulates estrogen biosynthesis. Polymorphisms in *CYP19A1 *have been related to the pathogenesis of breast cancer (BC). Inhibition of aromatase with letrozole constitutes the best option for treating estrogen-dependent BC in postmenopausal women. We evaluate a series of polymorphisms of *CYP19A1 *and their effect on response to neoadjuvant letrozole in early BC.

**Methods:**

We analyzed 95 consecutive postmenopausal women with stage II-III ER/PgR [+] BC treated with neoadjuvant letrozole. Response to treatment was measured by radiology at 4^th ^month by World Health Organization (WHO) criteria. Three polymorphisms of *CYP19A1*, one in exon 7 (rs700519) and two in the 3'-UTR region (rs10046 and rs4646) were evaluated on DNA obtained from peripheral blood.

**Results:**

Thirty-five women (36.8%) achieved a radiological response to letrozole. The histopathological and immunohistochemical parameters, including hormonal receptor status, were not associated with the response to letrozole. Only the genetic variants (AC/AA) of the rs4646 polymorphism were associated with poor response to letrozole (p = 0.03). Eighteen patients (18.9%) reported a progression of the disease. Those patients carrying the genetic variants (AC/AA) of rs4646 presented a lower progression-free survival than the patients homozygous for the reference variant (p = 0.0686). This effect was especially significant in the group of elderly patients not operated after letrozole induction (p = 0.009).

**Conclusions:**

Our study reveals that the rs4646 polymorphism identifies a subgroup of stage II-III ER/PgR [+] BC patients with poor response to neoadjuvant letrozole and poor prognosis. Testing for the rs4646 polymorphism could be a useful tool in order to orientate the treatment in elderly BC patients.

## Background

After cessation of ovarian activity at postmenopause, the aromatase [cytochrome P450 19 (CYP19); OMIM:107910] from bones, adipose tissue and muscle, becomes the key enzyme in estradiol and estrone biosynthesis through the aromatization of testosterone and androstenedione respectively [1]. Aromatase is encoded by *CYP19A1 *which maps at chromosome 15q21.1 and has a complex structure with a region that contains 10 tissue-specific non-coding upstream exons with separate promoters that regulate transcription in different cells and tissues [2]. Elevated levels of aromatase expression have been observed in breast tumors relative to normal breast tissue [3], and alterations in aromatase expression have been implicated in the pathogenesis of estrogen-dependent diseases, including breast cancer (BC) [4,5].

The major biological characteristic of BC is that two thirds of cases express estrogen (ER) and/or progesterone receptors (PgR) [2,6,7], endocrine manipulation being an effective treatment for these patients. Tamoxifen, a selective ER modulator, has for decades been the gold standard therapy in ER/PgR [+] BC patients [8]. However, in postmenopausal patients, aromatase inhibitors like letrozole have consistently demonstrated to be more effective than tamoxifen [9]. Letrozole is highly specific for the aromatase enzyme and inhibits whole-body aromatization of almost 99%, providing a highly and essentially complete withdrawal of estrogen in postmenopausal women [10].

Neoadjuvant hormonal therapy is a safety option for postmenopausal women with large operable or locally advanced hormone sensitive BC, increasing the possibilities of breast-conserving surgery (BCS) [11]. In this context, letrozole has shown to be more effective than tamoxifen, obtaining higher response and BCS rates following 4 months of preoperative therapy [12,13]. However, less than 60% of patients will respond to this optimal endocrine therapy [14]. To date, only the presence and intensity of hormonal receptors (ER/PgR) are useful tools as predictive markers in clinical practice. Identification of more accurate markers for more efficient patient selection to exclude non-responsive cases remains crucial.

Genetic polymorphisms in the *CYP19A1 *gene have been associated with altered sex hormone levels in serum and urine [15-17], providing an explanation for an elevated risk for BC in relation to estrogen exposure. For example, the tetranucleotide repeat polymorphism in intron 4 (TTTA)n has been associated with BC risk in initial studies [18-22]. The polymorphism rs10046 in the 3' untranslated region (3'-UTR) also showed inconsistent associations with BC risk [23-26] and has also been related with tumor stage [25], circulating sex hormone levels [15], HER2 status and disease free and overall survival [27,28]. rs4646 has been reported to be associated with circulating steroid hormone levels [17] and, as in rs10046, with the HER2 status of the tumor [27]. Hence, it is biologically plausible that the polymorphisms in the *CYP19A1 *gene may be associated with the response to aromatase inhibitors. Indeed, recently the rs4646 *CYP19A1 *polymorphism has been associated with letrozole efficacy in advance disease [29]; however, no evidence between *CYP19A1 *polymorphisms and therapeutic efficacy of aromatase inhibitors in early stage BC has yet been established.

In the present study, we perform a genetic analysis of three *CYP19A1 *polymorphisms in a series of postmenopausal endocrine-sensitive BC patients treated with neoadjuvant letrozole and describe their association both with radiological response at 4 months (RR4M) of treatment and with progression free survival (PFS).

## Methods

### Patient selection and DNA samples

We identified a total of 153 postmenopausal patients treated in our institution with letrozole as primary systemic therapy for stage II - III BC between April 1999 and March 2008. Only those cases which met the following criteria were eligible for the study: 1) histological diagnosis of invasive BC by tru-cut or biopsy; 2) positive hormonal receptors status measured by immunohistochemistry (> 10% ER and/or PgR); 3) preserved tumor block; 4) bidimensionally measurable disease by mammogram and/or breast ultrasound; 5) adequate radiological follow-up at 4 months (± 14 days); 5) treatment with letrozole (Femara: Novartis Pharma AG, Basel, Switzerland) as neoadjuvant therapy by 2.5 mg/day for a minimum of 4 months in the absence of progression or side effects; and 6) patients able to provide a blood sample.

Finally, 95 patients fulfilled all criteria, were contacted by their physician (AGZ, ALC, ARS, JG, MAC, VG) and agreed to participate on the study. All patients gave their written consent for the use of their DNA for this specific research proposal. In addition, this study was reviewed and approved by the FIVO Science and Ethics Committees.

Peripheral blood samples (7 ml) were obtained from these patients and processed for DNA extraction at the Laboratory of Molecular Biology.

Response to treatment at four months was done using the WHO criteria: complete response (CR) required the complete disappearance of all disease, partial response (PR) was defined as a reduction of ≥ 50% in tumor volume and stable disease (SD) was any reduction < 50% in tumor volume. The response evaluation was done by an independent radiologist (JCD).

Pre-treatment study included patient characteristics, menopausal status, disease history, histological grade and diagnosis, evaluation of hormone receptor status, HER2 and Ki67 status by immunohistochemical analysis on core-cut biopsies, ECOG performance status and tumor assessment. The clinical data were prospectively reviewed and stored within a specific database.

### Histological and immunohistochemical analysis

Core-cut biopsies were taken with a 14-guage needle before treatment and fixed in neutral buffered formalin for histological studies. The breast tumors were graded according to the modified Bloom and Richardson score on H&E-stained slides [30].

Four-micrometer sections from embedded blocks were cut on poly-L-lysine-coated slides, and dewaxed, endogenous peroxidase was inhibited with 3% hydrogen peroxide for 30 min. Immunoreactivity was enhanced with antigen retrieval treatment by heating the slides in a microwave oven for 10 min (700 W) in 10 mM sodium citrate buffer pH 7, followed by cooling for 20 min at room temperature. Sections were blocked with 20% horse serum in phosphate-buffer saline (PBS) and incubated with primary antibody for 1 h at room temperature. The incubation time for the secondary antibody and avidin-biotin complexes was 30 min at room temperature. Sections were extensively washed and the immunoreactions developed using DAB (0.05% 3'3' diamino-benzidine in 0.1% hydrogen peroxide). Negative controls included substitution of the primary antibody by mouse ascites or PBS. Slides were counterstained in Mayer hematoxylin, dehydrated, and mounted.

ER and PgR expression in primary tumor was immunohistochemically evaluated using the anti-ER (clone 6F11) and anti-PgR (clone 1A6) antibodies (Novocastra Laboratories, Newcastle upon Tyne, England) at a dilution of 1:40 and 1:30 respectively. Ki67 expression was also determined using the anti-Ki67 (clone MIB1) antibody (Dako Corp., Carpinteria, CA) at a 1:50 dilution.

HER2 protein expression was immunohistochemically evaluated using the HercepTest Kit (Dako Corp., Carpinteria, CA) according to the manufacturer's protocol. The scoring system was as follows: 0, tumors with no or weak staining in less than 10% of the cells; ***+***, tumors with a faint or barely perceptible membrane staining in more than 10% of cells or with noncircumferential staining; ++, moderate circumferential membrane staining; +++, strong circumferential membrane staining. Scores 0 and + were considered as negative; scores ++ and +++ were considered as positive for HER2 overexpression [31].

### DNA isolation

Genomic DNA used for genotyping studies was isolated from peripheral blood mononucleated cells, previously separated by centrifugation with Histopaque^®^-1077 (SIGMA-ALDRICH, LTD Irvine, UK), using the UltraClean DNA Blood Isolation Kit (MO BIO, Carlsbad, CA, USA). DNA integrity was evaluated by the A260/A280 absorbance ratio with a Nanodrop-1000 (Thermo Scientific) spectrophotometer.

### CYP19A1 Genotyping

The single nucleotide polymorphisms (SNPs) rs10046 (A/G) and rs4646 (C/A) located at the 3'-untranslated region (3'-UTR) of *CYP19A1*, previously associated with letrozole efficacy in advanced BC [29], and the rs700519 (C/T) in the first base of codon 264 (Arg^264^Cys), related with low levels of aromatase activity [32], were evaluated. The rs700519 was analyzed by PCR amplification and direct sequencing. Briefly, amplification was carried out in a final volume of 25 μl containing 100 ng genomic DNA, 0.3 μM of forward (5'-CATGAAGTGTAGGGTC-TATGTAAT-3') and reverse (5'-GATCTTTACACACCTCTACACAGT-3') primers, 0.2 mM dNTPs (deoxyribonucleoside triphosphates), 2 mM MgCl_2_, 1× _Buffer II (Applied Biosystems, New Jersey, USA) and 1 unit AmpliTaq Gold (Applied Biosystems, New Jersey, USA). PCR conditions were 94°C for 10 minutes; 35 cycles with denaturation at 94°C for 30 seconds, annealing at 52°C for 30 seconds, and elongation at 72°C for 30 seconds; and a final extension step at 72°C for 10 minutes. PCR products were visualized on 1.5% agarose gels containing ethidium bromide and after UV radiation. Purified PCR products were then sequenced using BigDye Terminator v3.1 Cycle Sequencing Kit (Applied Biosystems, Warrington WA1 4SR, UK) and electrophoresed on a ABI 3130xl Genetic Analyzer (Applied Biosystems). Sequencing analysis was performed with the Sequencing Analysis 5.2 Software (Applied Biosystems).

Genotyping of the rs10046 and rs4646 polymorphisms were carried out by allelic discrimination using specific TaqMan SNP Genotyping Assays and following the manufacturer's instructions. In brief, 20 μl PCR reactions were carried out containing 10.0 μl TaqMan Universal PCR Master Mix (Applied Biosystems, New Jersey, USA), 20.0 ng DNA template and 1.0 μL TaqMan SNP Genotyping Assays (Applied Biosystems, Foster City CA 94404). The references of selected assays were: C_8234731_1 (rs10046) and C_8234730_1 (rs4646). All assays were performed in 96-well plates including non-template controls. PCR reactions were read on a 7500 Fast Real Time PCR System in end-point mode using the Allelic Discrimination Sequence Detector software (Applied Biosystems).

### Statistical analysis

Statistical analysis was carried out using the SPSS statistical software package (version 15.0.1, SPSS Inc., Chicago, Illinois, USA). A total of 95 patients were included in the analysis. Data on radiological response at the fourth month of induction therapy, progression and survival status, immunohistochemical markers, histological grade, stage and genotype were available for these patients.

For the statistical analysis we used binary variables reflecting the positivity status of the measures (yes or no) as well as the genotypic status of each polymorphism (homozygous reference or heterozygous/homozygous less frequent). Association with histopathological parameters, all categorical, was also assessed using a chi-square test to determine homogeneity or linear trend for ordinal variables. The significance level was set at 5%.

Deviations of genotype frequencies from those expected under Hardy-Weinberg equilibrium (HWE) were assessed by χ^2 ^(p > 0.05) tests [33].

To study the impact of the biological factors on progression-free survival (PFS), the Kaplan-Meier proportional risk test (Log Rank) was used [34,35]. PFS was defined as the time interval from the start of treatment to local or distant disease progression. Those patients that reported disease progression during the follow-up were considered as censored, being the data of confirmation of this progression considered in the calculation of PFS. Univariate predictors of PFS were entered into a Cox proportional hazards model using stepwise selection to identify the independent predictors of poor outcome, a confidence interval (CI) of 95% was also considered [36].

## Results

### Patient and tumor characteristics

Ninety-five stage II-III ER/PgR-positive BC in postmenopausal women treated with letrozole as neoadjuvant therapy were analyzed. The median age of the series was 78.3 years (range: 61.7-89.8). The histopathological and clinical characteristics of the series are summarized in Table 1. As shown, 67 patients presented infiltrating ductal carcinoma (IDC), 16 infiltrating lobular carcinoma (ILC) and the remaining 12 cases corresponded to 9 mucinous carcinomas, 2 tubular carcinomas and one medullar carcinoma. The histological grade was available in only 52 of the IDC due to sample limitations. Most of the cases were stage IIa BC (68.4%), the majority of cases having a T2 tumor (80%). All tumors expressed HR (ER or PgR or both). Considering the whole HR expression for each patient we distinguished two groups of cases: those having low level HR (expressing HR in ≤ 40% of tumor cells) and those with a high HR expression (> 40% of cells). Ten cases were classified in the first group (10.5%). HER2 and Ki67 expression was also evaluated, 32 cases were HER2 ++/+++ (33.7%) and 32 out of 73 cases analyzed expressed Ki67 (44.4%).

**Table 1:** Clinical and pathological characteristics of the patients.

Parameter	n (%)
Histological diagnosis*	
IDC	67 (70.5)
ILC	16 (16.8)
Others	12 (12.6)
**Histological grade**	
I	29 (55.8)
II	19 (36.5)
III	4 (7.7)
**Tumor size (T)**	
T2	76 (80.0)
T3-T4	19 (20.0)
**Lymph node involvement (N)**	
N0	76 (80.0)
N1-2	19 (20.0)
**Stage**	
IIa	65 (68.4)
IIb	20 (21.1)
IIIa-IIIb	10 (10.5)
**ER**	
Negative	5 (5.3)
10-40%	8 (8.4)
40-70%	25 (26.3)
> 70%	57 (60.0)
**PgR**	
Negative	28 (29.5)
10-40%	18 (18.9)
40-70%	24 (25.3)
> 70%	25 (26.3)
HR**	
≤ 40%	10 (10.5)
> 40%	85 (89.5)
**HER2**	
Negative	39 (41.1)
+	24 (25.3)
++	22 (23.2)
+++	10 (10.5)
**Ki67**	
Negative	40 (55.6)
Positive	32 (44.4)
**Response to letrozole**	
Non responders	60 (63.2)
Responders	35 (36.8)
**Progression status**	
No progression	77 (81.1)
Progression	18 (18.9)
**Surgery status**	
No surgery	30 (32.0)
Surgery	65 (68.0)

*IDC, Infiltrating ductal carcinoma; ILC, Infiltrating lobular carcinoma; Others (Mucinous, tubular and medullar carcinomas).

**Hormonal status was considered as ≤ 40% when either estrogen and progesterone receptors were ≤ 40%, and as > 40% when at least one of the receptors were > 40%.

The median treatment period with letrozole as neoadjuvant therapy was 10.23 months (range: 3.53-85). Response to treatment was evaluated by radiological means (mammogram or ultrasound) at four months of letrozole administration using WHO criteria [37]. A total of 34 partial responses (PR) and one complete response (CR) where achieved, and were classified as responders for the analysis. Hence, the overall radiological response rate at 4 months (RR4M) was 36.8%. On the contrary, 55 women remained with stable disease (SD) and 5 developed progression disease (PD), these all being considered as non-responders.

### CYP19A1 polymorphism analysis

A total of three single nucleotide polymorphisms (SNPs) were evaluated: one located in exon 7 (rs700519) and two in the 3'-UTR region of *CYP19A1 *(rs10046 and rs4646). Polymorphism references, genotypes, gene and chromosome locations, and frequency distribution of observed genotypes in the series are shown in Table 2. In addition, values for Hardy-Weinberg (HW) [33] equilibrium were estimated for each polymorphism and are also listed in Table 2. Both rs10046 and rs4646 were in HW equilibrium; however, for rs700519 this calculation was not possible as no genetic variant (CT, TT) was observed in any of the analyzed cases.

**Table 2:** *CYP19A1 *polymorphisms and genotype frequencies

db-SNP id	Genotype	n (%)	p*	Gene location	Chromosome position**
**rs700519**	CC	89 (100)	-	Exon 7 (codon 264)	49.295.260
	CT	0 (0)			
	TT	0 (0)			
**rs10046**	AA	23 (24.2)	0.127	3'UTR	49.290.278
	AG	57 (60.0)			
	GG	15 (15.8)			
**rs4646**	CC	57 (60.0)	0.397	3'UTR	49.290.136
	AC	30 (31.6)			
	AA	8 (8.4)			

*p > 0.05 is consistent with the Hardy-Weinberg equilibrium; ** Sequence of reference NT_010194.16.

### Analysis between RR4M and clinical, pathological and genotypic parameters

No statistically significant association was found between RR4M and the clinical and pathological parameters (Table 3). However, an overrepresentation of the genetic variants (AC/AA) of the rs4646 polymorphism was observed in the non-responder group (48.3%) compared with the responders (25.7%) (p = 0.03) (Table 3).

**Table 3:** Association of clinical, pathological and genotypic parameters with radiological response at 4^th ^month of letrozole administration.

Parameters	Responders (%)	Non-responders (%)	p
**Age **(years)			
≤ 78.3	19 (54.3)	29 (48.3)	0.576
> 78.3	16 (45.7)	31 (51.7)	
**Histological diagnosis**			
IDC	25 (71.4)	42 (70.0)	0.843
ILC	5 (14.3)	11 (18.3)	
Others	5 (14.3)	7 (7.4)	
**Histological grade**			
I	13 (61.9)	16 (51.6)	0.606
II	6 (28.6)	13 (41.9)	
III	2 (9.5)	2 (6.5)	
**Tumor size (T)**			
T2	31 (88.6)	45 (75.0)	0.111
T3-T4	4 (11.4)	15 (25.0)	
**Lymph node involvement (N)**			
NO	28 (80.0)	48 (80.0)	1.000
N1-2	7 (20.0)	12 (20.0)	
**Stage**			0.546
IIa	26 (74.3)	39 (65.5)	
IIb	7 (20.0)	13 (21.7)	
IIIa-IIIb	2 (5.7)	8 (13.3)	
**ER**			
≤ 40%	3 (8.6)	10 (16.7)	0.268
> 40%	32 (91.4)	50 (83.3)	
**PgR**			
≤ 40%	15 (42.9)	31 (51.7)	0.407
> 40%	20 (57.1)	29 (48.3)	
**HR**			
≤ 40%	2 (5.7)	8 (13.3)	0.243
> 40%	33 (94.3)	52 (86.7)	
**HER2**			
0, +	21 (60.0)	42 (70.0)	0.320
++, +++	14 (40.0)	18 (30.0)	
**Ki67**			
Negative	15 (55.6)	25 (55.6)	1.000
Positive	12 (44.4)	20 (44.4)	
**rs10046**			
**GG**	6 (17.1)	9 (15.0)	0.782
**AG/AA**	29 (82.9)	15 (85.0)	
**rs4646**			
**CC**	26 (74.3)	31 (51.7)	**0.030**
**AC/AA**	9 (25.7)	29 (48.3)	

### Clinical, pathological, genotypic parameters and progression free survival (PFS)

**Figure 1:**
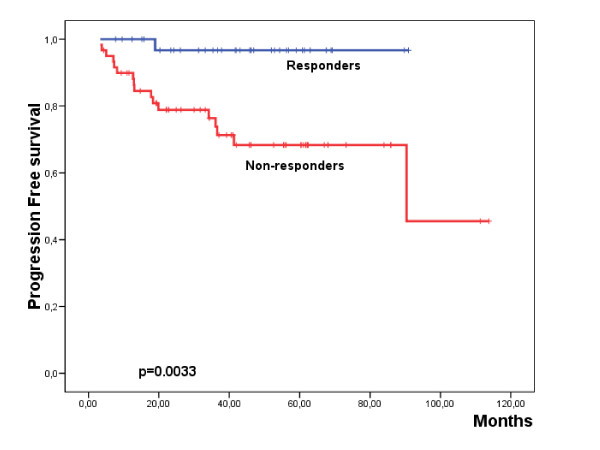
**Kaplan Meier plot showing the progression-free survival with regard the radiological response to letrozole at 4^th ^month (RR4M) of the induction therapy**.

**Figure 2:**
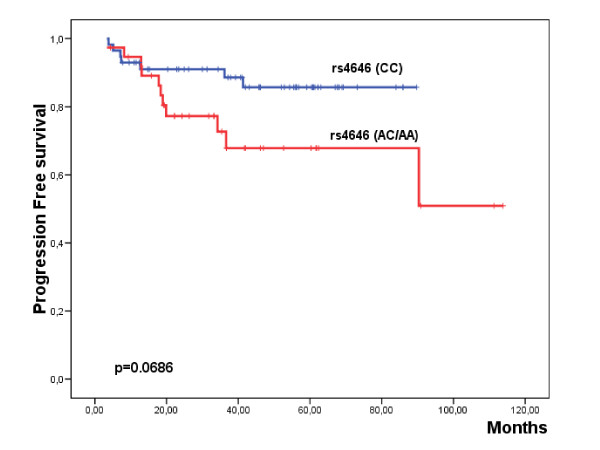
**Kaplan Meier plot showing the progression-free survival with regard the genetic variants of rs4646 in the global series**.

The median follow-up of the series was of 40.6 months (range: 4.3-113.8 months). A total of 18 patients (18.9%) reported disease progression during this period (Table 1). Among the analyzed parameters, IIIa-IIIb stages and the non response to letrozole induction therapy were significantly associated with shorter PFS (Table 4; Additional file 1: Supplemental Table S1; Fig. 1). Interestingly, those patients carrying the genetic variants (AC/AA) of rs4646 presented a worse PFS than the patients homozygous for the reference variant (Table 4; Additional file 1: Supplemental Table S1; Fig. 2). Multivariate analysis showed that lymph node involvement [Hazard Ratio (HR) = 4.3 (1.7-11); p = 0.002] and RR4M [HR = 11 (1.5-83); p = 0.012] constituted independent prognostic factors for PFS.

**Table 4:** Association of clinical, pathological and genotypic parameters with progression free survival (PFS).

Parameters	n	Events	%PFS	p
**Tumor size**				
T2	76	12	62.6	0.089
T3-T4	19	6	59.3	
**Lymph node involvement**				
N0	76	10	57.31	0.0011
N1-N2	19	8	44.7	
**Stage**				
IIa	65	8	58.2	0.0232
IIb	20	6	65.1	
IIIa-IIIb	10	4	42.3	
**RR4M**				
Responders	35	1	96.7	0.0033
Non-responders	60	17	45.6	
**rs10046**				
GG	18	3	80.0	0.967
AG/AA	80	15	81.3	
**rs4646**				
CC	57	7	85.7	0.0686
AC/AA	38	11	50.9	

### Clinical, pathological, genotypic parameters and PFS according to the surgery status

Sixty-five out of the 95 patients (68%) underwent surgery after letrozole induction therapy and no association was observed between the progression status and surgery (Table 1). In fact, 20% of non-surgery cases progressed, while 18.5% of the surgery group experienced progression events (p = 0.861). In addition, no significant differences in terms of PFS were observed between the non-surgery (74.5%) and surgery (60.4%) groups (p = 0.600). However, when we compared clinical, pathological and genetic parameters between the two groups of patients according to surgery status, we observed that 77% of patients of the non-surgery group were over the median age, compared with 37% of the patients from the surgery group (p = 0.00032). Moreover, rs4646 variant (AC/AA) was overrepresented in the non-surgery group (57%) in contrast to the surgery group (32%) (p = 0.024).

**Figure 3:**
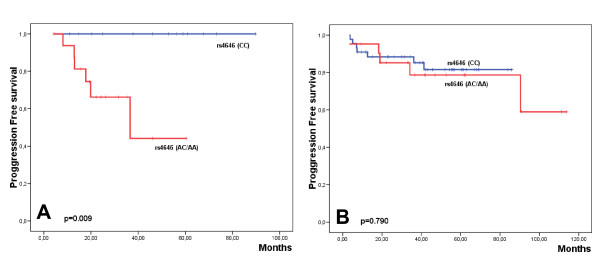
**Kaplan Meier plot showing the progression-free survival with regard the genetic variants of rs4646 in the no surgery (A) and surgery group of patients (B)**.

Univariate analysis performed for PFS for each group of patients revealed that in the non-surgery group, RR4M and r4646 where associated with a worse prognosis (Table 5; Additional file 2: Supplemental Table S2). Indeed, the 6 patients who progressed were genetically characterized by the variant rs4646 (AC/AA) (Fig. 3A). However, in the multivariate analysis only the polymorphism rs4646 emerged as an independent prognostic factor although it did not reach statistical significance (p = 0.191).

**Table 5:** Association of clinical and genotypic parameters with progression free survival (PFS) according to surgery status.

	No surgery				Surgery			
**Parameters**	**n**	**Events**	**%PFS**	**p**	**n**	**Events**	**%PFS**	**p**
**Ki67**								
Negative	17	5	66.9	0.686	23	1	50.0	0.023
Positive	5	1	80.0		27	8	69.6	
**RR4M**								
Responders	10	0	100	0.041	25	1	95.2	0.030
Non-responders	20	6	58.8		40	11	47.8	
**rs4646**								
CC	13	0	100	0.009	44	7	81.5	0.790
AC/AA	17	6	44.1		21	5	59.0	

In the group of patients operated after letrozole induction, only Ki67 expression and RR4M were associated with worse PFS (Table 5; Additional file 2: Supplemental Table S2), although none of these variables were independent indicators of poor outcome. Contrary to that observed in the non-surgery group, no association of the rs4646 variants with PFS was observed (Fig. 3B).

## Discussion

We describe a relationship between the genetic variants (AC/AA) of the rs4646 polymorphism of *CYP19A1 *and a poor response to neoadjuvant treatment with letrozole in postmenopausal women with ER/PgR [+] BC. The radiological response was measured at the 4^th ^month of letrozole induction, the observed response rate being 36.8%, similar to the radiological response reported in the P024 trial [12].

At genetic level, mutations in the aromatase gene may lead to a functionally less sensitive aromatase phenotype. Although mutations in the *CYP19A1 *gene can be generated *in vitro*, to date no somatic mutation has been found in clinical samples [38]. However, several genetic polymorphisms of *CYP19A1 *have been reported so far, although the possible functional significance of most of these remains undefined. In the case of BC, population-based studies of common *CYP19A1 *polymorphisms have generated inconsistent results with regard to their possible association with sex hormone levels, cancer risk, HER2 status or survival [15,19,21,27,28]. No association between the HER2 or hormonal status of either rs10046 or rs4646 was observed in our series.

Whether aromatase inhibitor agents interact with the different *CY19A1 *genotypes has not so far been clearly established. Only three previous studies have attempted to demonstrate the effect of certain polymorphisms in *CYP19A1 *on the efficacy of aromatase inhibitors. In the first, Fasching et al., in a series of patients treated with hormonal therapy for more than 2 years, did not observe significant differences between the rs700519, rs10046 and rs4646 polymorphisms with therapy. Unfortunately, the type of antihormonal therapy was not recorded for this analysis [27]. In the second study, a population-based and *in vitro *study, Ma et al. revealed reductions in the functional activity of aromatase for four phenotypes resulting from non-synonymous changes [rs700519 (Arg^264^Cys), rs28757184 (Thr^201^Met), rs2236722 (Trp^39^Arg), and rs56658716 (Met^364^Thr)] [32]. These authors found that levels or aromatase enzyme activity decreased dramatically for the Thr^364^, and also observed a slight decrease in Cys^264 ^allozyme activity. They also demonstrated that the mechanism by which non-synonymous SNPs affect the enzymatic activity is a consequence of an alteration in the enzyme protein level [32]. In the same study, these authors reported that these variants had no significant differences in their affinity to the aromatase inhibitors letrozole and exemestane [32]. Interestingly, the polymorphism rs700519 has also been reported as a prognostic factor in a Chinese study, mainly in a subgroup of premenopausal women [28], but not in the Caucasian population [27]. In addition, it is important to note the differences between these two populations regarding the minor allele frequency of rs700519, being only 3.2% for the Caucasian population (1,257 patients) [27], in comparison with 15.1% in the Asian Cohort (1,136 patients) [28]. In our series, the genetic variants of this polymorphism were underrepresented and not found in any of the cases.

The third study evaluated the efficacy of treatment with letrozole in advanced hormone receptor-positive BC patients with respect to two polymorphisms located at the 3'-UTR (rs10046 and rs4646) and one in intron 2 [rs727479 (G/T)] of the *CYP19A1 *[29]. The authors found that genetic variants of rs4646 were associated with a greater efficacy of letrozole in terms of time to progression. Hence, patients with the variant genotype (AC/AA) had a three times greater time to progression than the patients with the reference genotype (CC). Furthermore, these authors reported that the frequency of the variant alleles for rs4646 was significantly higher in the responder (61%) than in the non-responder group of patients (40%) [29]. These observations clearly differ from those reported in our series, the genotypic variants of rs4646 being more frequently represented in the non-responder group to letrozole after 4 months of induction therapy (48% vs. 26%). In this regard, there are two main issues that could explain the differences between these two studies. First, and independently of the disease stage, are the criteria for patient selection. Whereas in our series the patients were treated with letrozole as a first line, in the study of Colomer et al. the patients had progressed from a previous treatment with tamoxifen [29]. It is well established that tamoxifen metabolism is influenced by the number of mutant alleles of the gene encoding cytochrome P450 2D6 (*CYP2D6*), because this enzyme affects the levels of endoxifen, the active tamoxifen metabolite. Thus, for patients who are wild type for *CYP2D6*, the 5-year disease-free survival outcomes are similar to or perhaps even superior with tamoxifen than with aromatase inhibitors [39]. Therefore, previous treatment with tamoxifen could have genetically selected a population of patients more sensitive to letrozole. Secondly, in our study we used constitutive DNA obtained from peripheral blood for the genetic analysis, whereas Colomer et al. performed the genetic study on DNA obtained from the fixed and paraffin-embedded tumors [29]. The polymorphism studies performed on tumor samples are subject to genetic alterations that can affect the chromosomal region of the gene under study. In this way, *CYP19A1 *is located in the 21.2 region of the long arm of chromosome 15 (15q21.2) [40], and this region has been reported to be a frequent target of allelic imbalance in advanced breast carcinomas [41], and which could affect the frequency distribution of the allelic variants.

From our series, 65 women underwent surgery after letrozole induction, being age the only difference observed between the operated and non-operated patients. In fact 77% of patients within the non-surgery group were over 78.3 years. We have demonstrated that genetic variants of rs4646 have prognostic value, especially in this group of patients. Indeed, the 6 women who progressed within the group of non-surgery patients were genetically characterized by the variant rs4646 (AC/AA). In this elderly group of patients the decision for undergoing surgery is not always easy and very often both the patient and the clinician opt for a local control of the tumor maintaining the treatment with letrozole. Hence, in this group of patients, in which the tumor is not removed, is where the rs4646 polymorphism identifies the women who progressed, indicating, and despite the small number the cases included in our series, that women with the rs4646 (AC/AA) genotype could benefit from another therapeutic approach.

The regulatory interactions between the ER, growth factor receptors and other kinase signalling pathways could also determine the response to endocrine therapy. In the adjuvant setting, several studies suggest that patients overexpressing HER2 may derive relatively less benefit from endocrine therapy [38]. In the P024 trial, ER [+] tumors that were also EGFR and/or HER2 [+] responded significantly better to letrozole than to tamoxifen (88% vs 21% respectively, P = 0.0004) [13]. The IMPACT study, that confronted tamoxifen with anastrozole, or the preoperative combination of both, observed a similar effect in favour of anastrozole for HER2 [+] tumors (Odds Ratio 58% vs. 22%), although not reaching statistical significance [42]. In our series, as in both randomized trials, no differences in activity were observed relating to HER2 expression among the patients receiving letrozole.

Biological studies of tissues obtained during neoadjuvant therapy have thrown a different perspective on this issue. Short-term estrogen deprivation leads to profound changes in transcriptional profiles, and these changes can be used as predictive tools. Tumor expression of the proliferation antigen Ki67 after 2 weeks of endocrine treatment (tamoxifen or anastrozole) predicts for clinical response and recurrence-free survival [42]. However, among cases on anastrozole, only 7% did not present a reduction in the Ki67 expression at 2 weeks. In many cases, this reduction in the tumor proliferation rate is only modest and could not be sufficient to determine a resistance to the treatment. As expected, early tumor changes are not limited to proliferation markers, but to other genes directly regulated by ER, including the aromatase itself [43]. Other, currently ongoing, approaches have used gene expression profiling techniques able to predict response to endocrine therapy, but the results obtained so far are inconsistent [43]. In our series, expression of proliferating marker Ki67 constituted a factor of poor prognosis in the group of patients undergoing surgery following letrozole induction therapy, suggesting that this marker could identify tumors with a more aggressive behavior.

## Conclusion

We have found that genetic variants of the polymorphism rs4646 in the 3'-UTR of *CYP19A1 *are associated to poor response to letrozole after 4 months induction therapy and to poor outcome of elderly patients without surgery after letrozole administration in postmenopausal women with ER/PgR [+] BC. Although our study would suggest that analysis of rs4646 could improve the clinical management of these patients by facilitating a more individualized therapy, further studies based on larger series are necessary to confirm these findings.

## List of abbreviations

3'-UTR: 3'-untranslated region; *CYP19A1*: aromatase gene; BC: breast cancer; BCS: breast conserving surgery; CR: complete response; DAB: diamino-benzidine; ER: estrogen recetor; HWE: Hardy-Weinberg equilibrium; IDC: infiltrating ductal carcinoma; ILC: infiltrating lobular carcinoma; PR: partial response; PBS: phosphate-buffer saline; PgR: progesterone receptor; PD: progression disease; PFS: progression free survival; RR4M: radiological response at 4 months; SNPs: single nucleotide polymorphisms; SD: stable disease; WHO: World Health Organization.

## Competing interests

The authors declare that they have no competing interests.

## Authors' contributions

ZGC carried out the molecular genetic studies, participated in the genetic analysis and drafted the manuscript. AGZ reviewed the clinical records of the patients included in the study and drafted the manuscript. AFS performed the genetic analysis. ALlC, ARS, JG, MAC and VG treated most of the patients included in the study and updated the clinical follow-up. AC, SA and ALlB carried out the histopathological and immunohistochemical analysis. JCD performed the radiological evaluation of the study. JC and CVA performed the trucut biopsies and informed to the patients about the study. JALG conceived of the study, performed the statistical analysis and participated in its design and coordination and helped to draft the manuscript. All authors read and approved the final manuscript.

## Pre-publication history

The pre-publication history for this paper can be accessed here:

http://www.biomedcentral.com/1471-2407/10/36/prepub

## Supplementary Material

Additional file 1 **Table S1. **Association of clinical, pathological and genotypic parameters with progression free survival (PFS). This table shows the associations of the clinicopathological and genetic parameters with PFS that have not been statistically significant. Click here for file

Additional file 2 **Table S2.** Association of clinical, histopathological and genotypic parameters with progression free survival (PFS) according to surgery status. This table shows the associations of the clinicopathological and genetic parameters with PFS that have not been statistically significant according to surgery status. Click here for file
