# Supplementary material for: A polymorphism at the 3'-UTR region of the aromatase gene defines a subgroup of postmenopausal breast cancer patients with poor response to neoadjuvant letrozole
Source: BMC Cancer. 2010 Feb 9;10:36. doi: 10.1186/1471-2407-10-36 (PMC2830181; doi:10.1186/1471-2407-10-36)
Supplement: Additional file 1 — Table S1. Association of clinical, pathological and genotypic parameters with progression free survival (PFS). This table shows the associations of the clinicopathological and genetic parameters with PFS that have not been statistically significant. [file 1471-2407-10-36-S1.DOC]

**Table S1.** Association of clinical, pathological and genotypic parameters with progression free survival (PFS).

| **Parameters** | **n** | **Events** | **%PFS** | **p** |
| --- | --- | --- | --- | --- |
| **Age(years)**  ≤78.3  >78.3 | 48  47 | 10  8 | 59.8  75.8 | 0.8554 |
| **Histological diagnosis**  IDC  ILC  Others | 67  16  12 | 12  4  2 | 77.4  40.4  82.5 | 0.948 |
| **Histological grade**  I  II  III | 29  19  4 | 3  6  1 | 81.5  66.1  66.7 | 0.261 |
| **ER**  **≤**40%  >40% | 13  82 | 4  14 | 68.4  53.5 | 0.2131 |
| **PgR**  **≤**40%  >40% | 46  49 | 11  7 | 49.1  83.5 | 0.303 |
| **HR**  **≤**40%  >40% | 10  85 | 3  15 | 68.6  53.3 | 0.329 |
| **HER 2**  0,+  ++,+++ | 63  32 | 13  5 | 38.9  81.04 | 0.5115 |
| **KI 67**  Negative  Positive | 40  32 | 6  9 | 43.11  70.9 | 0.2103 |
