# Supplementary material for: A polymorphism at the 3'-UTR region of the aromatase gene defines a subgroup of postmenopausal breast cancer patients with poor response to neoadjuvant letrozole
Source: BMC Cancer. 2010 Feb 9;10:36. doi: 10.1186/1471-2407-10-36 (PMC2830181; doi:10.1186/1471-2407-10-36)
Supplement: Additional file 2 — Table S2. Association of clinical, histopathological and genotypic parameters with progression free survival (PFS) according to surgery status. This table shows the associations of the clinicopathological and genetic parameters with PFS that have not been statistically significant according to surgery status. [file 1471-2407-10-36-S2.DOC]

**Table S2.** Association of clinical, histopathological and genotypic parameters with progression free survival (PFS) according to surgery status.

|  | **No surgery** | | | | | **Surgery** | | | |
| --- | --- | --- | --- | --- | --- | --- | --- | --- | --- |
| Parameters | **n** | **Events** | **%PFS** | **p** |  | **n** | **Events** | **%PFS** | **p** |
| **Age**  ≤ 78.3  > 78.3 | 7  23 | 3  3 | 51.4  80.4 | 0.066 |  | 41  24 | 7  5 | 63.4  72.5 | 0.541 |
| **Histological diagnosis**  IDC  ILC  Others | 17  7  6 | 4  1  1 | 64.1  83.3  80.0 | 0.873 |  | 50  9  6 | 8  3  1 | 80.6  38.9  83.3 | 0.742 |
| **Histological grade**  I  II  III | 5  5 | 1  1 | 50.0  75.0 | 0.808 |  | 24  14  4 | 2  5  1 | 85.6  63.5  66.7 | 0.164 |
| **Tumor size (T)**  T2  T3-T4 | 23  7 | 4  2 | 77.3  66.7 | 0.597 |  | 53  12 | 8  4 | 64.2  57.8 | 0.100 |
| **Lymph Node Involvement (N)**  N0  N1-N2 | 25  5 | 3  3 | 83.2  26.7 | 0.01 |  | 51  14 | 7  5 | 57.9  52.2 | 0.031 |
| **Stage**  IIa  IIb  IIIa-IIIb | 22  4  4 | 3  1  2 | 81.2  75.0  33.3 | 0.267 |  | 43  16  6 | 5  5  2 | 59.8  64.0  40.0 | 0.084 |
| **ER**  ≤40%  >40% | 2  28 | 1  5 | 50.0  76.6 | 0.154 |  | 11  54 | 3  9 | 72.7  54.7 | 0.379 |
| **PgR**  ≤40%  >40% | 15  15 | 4  2 | 65.5  82.5 | 0.370 |  | 31  34 | 7  5 | 51.5  83.7 | 0.534 |
| **HR**  ≤40%  >40% | 2  28 | 1  5 | 50.0  76.6 | 0.154 |  | 8  57 | 2  10 | 75.0  54.2 | 0.622 |
| **HER2**  0, +  ++, +++ | 22  8 | 4  2 | 80.5  60.0 | 0.651 |  | 41  24 | 9  3 | 38.6  85.9 | 0.328 |
| **rs10046**  GG  AG/AA | 6  24 | 1  5 | 80.0  74.3 | 0.829 |  | 9  56 | 2  10 | 74.1  54.3 | 0.880 |
